# Succinate: An initiator in tumorigenesis and progression

**DOI:** 10.18632/oncotarget.17734

**Published:** 2017-05-10

**Authors:** Ting Zhao, Xianmin Mu, Qiang You

**Affiliations:** ^1^ Department of Biotherapy, Second Affiliated Hospital, Nanjing Medical University, Nanjing, Jiangsu 210011, China; ^2^ Medical Center for Digestive Diseases, Second Affiliated Hospital, Nanjing Medical University, Nanjing, Jiangsu 210011, China; ^3^ Department of Immunology, Nanjing Medical University, Nanjing, Jiangsu 211166, China

**Keywords:** succinate, SDH, GPR91, tumorigenesis, HIF

## Abstract

As an intermediate metabolite of the tricarboxylic acid cycle in mitochondria, succinate is widely investigated for its role in metabolism. In recent years, an increasing number of studies have concentrated on the unanticipated role of succinate outside metabolism, acting as, for instance, an inflammatory signal or a carcinogenic initiator. Actually, succinate dehydrogenase gene mutations and abnormal succinate accumulation have been observed in a battery of hereditary and sporadic malignancies. In this review, we discuss the unexpected role of succinate and possible mechanisms that may contribute to its accumulation. Additionally, we describe how the high concentration of succinate in the tumor microenvironment acts as an active participant in tumorigenesis, rather than a passive bystander or innocent victim. Focusing on mechanism-based research, we summarize some targeted therapies which have been applied to the clinic or are currently under development. Furthermore, we posit that investigational drugs with different molecular targets may expand our horizon in anticancer therapy.

## INTRODUCTION

Succinate was purified from amber in 1546 by a German chemist [1], and it rapidly became a major topic in biochemistry and bioenergetics studies [2]. Researchers focused all their energy on its role in metabolism. Succinate dehydrogenase (SDH) is an enzyme complex that consists of SDHA, SDHB, SDHC and SDHD subunits, and catalyzes succinate into fumarate in the tricarboxylic acid cycle (TCA cycle) [3]. Random mutation of SDH subunits by hereditary or acquired influences will contribute to the abnormal accumulation of succinate in the cytosol.

Recently, there have been numerous publications on the previously ignored roles of succinate beyond metabolism, especially in signal transduction, reactive oxygen species (ROS) production, hypoxia inducible factor 1 (HIF-1) activation and stabilization, and G protein-coupled receptor-91 (GPR91) stimulation and downstream signaling pathway cascades, which are closely associated with inflammatory and carcinogenic progression. Mutations in SDH have been identified in specific cancers, both genetic and sporadic, such as familial paraganglioma/pheochromocytoma (PGL/PCC), renal carcinoma [4], thyroid cancer [5], ovarian cancer [6], neuroblastoma [7], gastrointestinal stromal tumor [8, 9], and even testicular seminoma [10]. Moreover, elevated concentrations of succinate have been detected in cancer patients [11].

How does succinate facilitate tumorigenesis and progression? Additionally, are there any effective targeting strategies to influence succinate signaling? In our opinion, accumulated succinate results in reprogramed metabolites, HIF-1 activation and stabilization, ROS production, tumor necrosis factor receptor-associated protein 1 (TRAP1) up-regulation that leads to SDH inhibition, NRF2 pathway activation and tumor-promoting inflammation, all these are indispensable elements in oncogenesis and tumor progression. In addition, we discuss some mechanism-based research and illustrate several theoretically feasible strategies which aim at making a small contribution to targeted therapies in the clinic.

### Roles of succinate in and outside metabolism

Succinate is an intermediate metabolite in mitochondria and is catalyzed into fumarate by succinate dehydrogenase (SDH) in the TCA cycle, which has been shown to play a crucial role in metabolism, such as electron delivery in the respiratory chain [12], substrate-level phosphorylation [13], ketone bodies utilization [14], haem metabolism [15], itaconate metabolism [16, 17] and the “GABA (γ-Aminobutyric acid) shunt” [18]. It has been studied as a mitochondrial respiration mediator and energy producer for more than 60 years, however, more and more researchers now focus on the broader roles of succinate outside metabolism [19]. It has been significantly associated with a series of special pathophysiological processes beyond metabolism, for example, contributing to the complications of specific metabolic diseases [1], promoting inflammatory activation reactions [18], oncogenesis and tumor progression [20].

We and others have demonstrated that succinate accumulation is commonly observed in a number of hereditary and sporadic malignancies, such as familial paraganglioma/pheochromocytoma (PGL/PCC) [21], thyroid cancer, ovarian cancer, neuroblastoma [7], gastric cancer and renal carcinoma [4]. Our data revealed that succinate accumulation promotes angiogenesis through GPR91-mediated STAT3 and ERK activation [22]. Recently, succinate was defined as a new “epigenetic hacker” [23, 24] to inhibit DNA and histone demethylases [25, 26], resulting in epigenetic alteration in carcinomas. Studies at both the biochemical and genetic levels have confirmed the status of succinate in cellular transformation and tumorigenesis [27, 28]. The mitochondrial chaperone, tumor necrosis factor receptor-associated protein 1 (TRAP1) [29] and nuclear ARRB1 [30] can trigger neoplastic growth, which relies on downregulating and inhibiting the activation of SDH. In contrast, deletion of TRAP1 to prevent SDH inhibition delayed prostatic tumorigenesis [31]. Taken together, these findings implicated succinate as the driver in tumor formation and progression.

### Succinate dehydrogenase and cancers link with SDH mutations

SDH is the key enzyme that converts succinate to fumarate in the TCA cycle and is composed of four subunits named SDHA, SDHB, SDHC and SDHD [32]. SDHA and SDHB are catalytic domains, while SDHC and SDHD are ubiquinone-binding and membrane-anchorage domains [3]. SDH is a key enzyme in the mitochondrial TCA cycle and integrates into the mitochondrial membrane. It also functions as an electron pumping complex in the electron transfer chain, the reaction in which reduced flavin adenine dinucleotide (FADH2) is generated. Generally, succinate is the connection between oxidative phosphorylation and electron transportation.

Mutations of the gene encoding SDH result in the accumulation of succinate. This leads to the metabolic reprograming of the “tumor microenvironment”, despite normal oxygen levels, providing an advantageous environment for tumor survival. Although succinate is known as a classic “housekeeping gene”, SDH mutations are commonly found in a series of neoplasms and different subunit mutations can lead to different types of tumors. It is noteworthy that most mediastinal paragangliomas (PGLs) were related to SDHD gene mutations [33], whereas germline mutations of SDHB and SDHC play a minor role in sporadic head and neck paraganglioma [34]. To date, the genomics research on PGL/PCC has demonstrated that mutations of SDHD and SDHC cause PGL1 and PGL3, while PGL4, PGL5, and PGL2 are associated with mutations in the large subunit genes SDHB, SDHA and SDHAF2, respectively [35]. In the case of renal cell carcinoma and papillary thyroid cancer, dysfunction of the SDHB domain is the greatest risk factor [36, 37]. Additionally, mutations in SDHA, SDHB, and SDHC have also been implicated in gastrointestinal stromal tumors [8, 38, 39].

### Factors responsible for succinate accumulation

As previously described, SDH mutations have been observed in some hereditary and non-genetic tumors, such as PGL/PCC, thyroid cancer and ovarian cancer. As it is a key enzyme involved in the TCA cycle, both inactivity and dysfunction of SDH can lead to succinate accumulation and low level of fumarate. SDH requires oxidized FAD^+^ and NAD^+^ as cofactors [40], while the cancer cells are deficient in these factors due to mitochondrial dysfunction. Mitochondrial respiratory disorder resulting from enzyme dysfunction has been shown to be directly responsible for the initiation of cancer, while mutations of SDH should bear most of the responsibility. Another significant protein responsible for succinate elevation is TRAP1, a mitochondrial chaperone which is highly expressed in a series of tumor cells [41]. TRAP1 inhibits respiratory complex II to downregulate the activity of SDH, thus leading to high concentrations of succinate.

**Figure 1:**
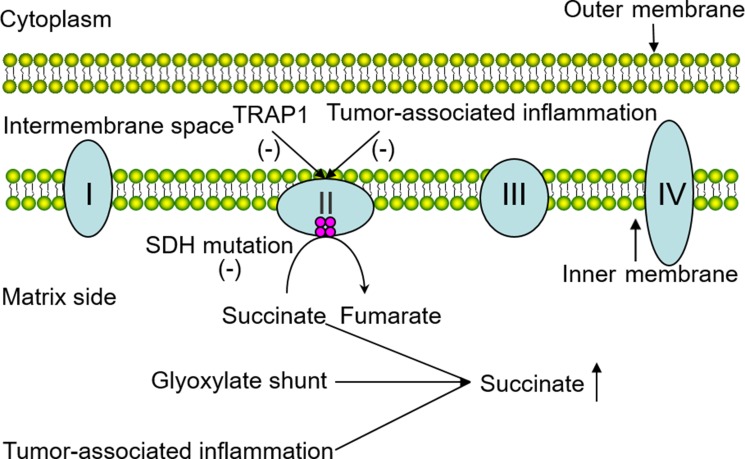
Possible factors responsible for succinate accumulation in the tumor The pivotal role of SDH which functions in electron delivery in the mitochondrial respiratory chain is intuitively illustrated above, and the involvement of SDH mutations, glyoxylate shunt and tumor-associated inflammatory response leading to succinate accumulation are shown in the diagram.

Several other possible elements also take charge of succinate accumulation in neoplastic tissues. Recent studies have shed some light on how succinate accumulates in various immune cells in inflammatory cascades. They suggested that the glyoxylate shunt which converts isocitrate to succinate via the enzyme isocitrate lyase (ICL) is responsible [18]. Previously, tumor formation and inflammatory response have been considered to be separate pathological processes. Until recent years, tumor-promoting inflammation has long been recognized as an enabling characteristic of cancer, and tumor-associated inflammation has been demonstrated in cancer. Hereby, we posit that inflammatory cells stationed in cancer tissues can release chemicals including succinate to favor neoplastic progression at the early stages. In a similar way, the tumor-associated inflammatory response can also decrease the activity of SDH. Although in this tumor condition, succinate can also be synthesized through physiological pathways separate from these pathological processes. For example, it can be derived from glutamine, fatty acid metabolism and the “GABA (γ-Aminobutyric acid) shunt” pathway. In summary, the involvement of SDH mutations, glyoxylate shunt and the tumor-associated inflammatory response can indeed contribute to high concentrations of succinate in cancer (Figure 1).

### Possible roles of succinate in tumorigenesis and progression

### Dysregulation and remodeling of mitochondrial function

As Otto Warburg had firstly proposed in the early 1920s that tumor cells can remodel their glucose metabolism, thus their energy metabolism, even in the presence of sufficient oxygen, resulting in energy production mainly through glycolysis, thereby bringing about a process intituled “aerobic glycolysis” [27, 42]. This theory may sound counterintuitive at first sight but was subsequently demonstrated to be true afterwards [43]. Reprogramming of the TCA cycle was a biological hallmark of cellular energetic adaptation to most effectively sustain high neoplastic proliferation, which subsequently underpinned the etiology of cancer during its multistep development [44].

Glycolytic fueling has been confirmed to be inextricably associated with oncogene activation (e.g., RAS, MYC) and tumor suppressor mutation (e.g., TP53, Rb) [45]. In normal cells, the oncogenes (including MYC) are down-regulated due to extracellular and intracellular cues, such as oxygen, to increase glutamine, glycolysis absorption and metabolism, and lactate production. When in a hypoxia or tumor condition, the ATP consumption will increase absolutely or relatively on demand [46]. The oncogenes are then activated or altered in order to enhance glycolysis and stimulate cellular biomass accumulation [47]. In addition to oncogene activation and tumor suppressor mutation, enzyme dysfunction in the TCA cycle also contributes to energy remodeling to some degree. A concise summary, all these adjustments resulting in succinate accumulation in cancer cells will conversely facilitate cellular transformation and tumor evolvement.

### Pseudohypoxia and HIF stabilization

Pseudohypoxia is a common feature in most advanced tumors though the oxygen content is normal, and much of the adaptions are modulated by a number of transcription factors such as Hypoxia inducible factor 1 (HIF-1) [30]. HIF-1 was first discovered by Semenza in 1992, and it was elucidated to be able to promote erythropoiesis through binding to the erythropoietin gene enhancer [48]. HIF-1 is a heterodimer consisting of an oxygen-labile α domain and a constitutively expressed β domain [49]. The α domain is the regulatory and active subunit of HIF-1 and is induced by a hypoxia signal. HIF-1α serves as a transcriptional regulator with a transactivation domain at its C-terminal end and a nuclear localization signal at the N-terminal end [50, 51].

Abnormally accumulated succinate in mitochondria which causes by inherited or somatic mutations in random subunits of SDH will be freely transported to the cytosol via the dicarboxylic acid translocator in the mitochondrial inner membrane and the voltage-dependent anion channel (VDAC/porin) in the mitochondrial outer membrane [20]. Elevated concentrations of succinate in the cytosol have been confirmed to inhibit HIF-1α prolyl hydroxylases (PHD) [20], which hereafter hydroxylate the highly conserved prolyl residues on HIF-1α. After being hydroxylated, the von Hippen-Lindau (pVHL) E3 ubiquitin ligase recognizes the HIF-1α domain, then inciting proteasomes degradation which is termed ubiquitination [52]. This so-called catabolism of HIF-1α can be inhibited upon PHD inactivation due to the presence of succinate in the cytosol, and PHD inactivation finally results in activation and stabilization of HIF-1α. HIF-1α can then bind to HIF response elements (HREs) in target genes, resulting in upregulation of many glycolytic enzymes that regulate energy metabolism, thereby improving vasomotor response, promoting cell proliferation and enhancing angiogenesis which are indispensable in tumor maturation and invasion [53].

### ROS production

Reactive oxygen species (ROS) are a number of oxyradicals derived from mitochondria and are involved in oxygen metabolism. An example of ROS is superoxide anion (O_2_^−^), which is increasingly associated with oxidative damage relating to plenty of pathological and physiological processes, including signal transduction [54], cell apoptosis [55] and gene mutagenesis [56].

Transgenic mouse fibroblasts transfected with SDHC loss-of-function can narrowly escape complex II enzyme activity reduction, ROS production and elevated DNA mutation [57]. Subsequent studies showed that any defects in SDHB, SDHC, or SDHD, but not SDHA, will disrupt complex II enzymatic activity in mitochondria. Dysfunction of mitochondria stemming from TCA cycle enzyme inactivation leads to ROS overproduction directly and indirectly. Once elevated in the cytosol, ROS can oxidize amino acid residues within fatty acids and proteins, and cause irreversible DNA damage and genomic instability, leading to carcinogenesis and tumorigenesis [58].

In addition to the oxidative stress pathway, ROS production resulting from SDH defects can also act as signal transduction messengers to stabilize HIF-1α through oxidizing Fe2^+^ to Fe3^+^, as Fe2^+^ is a critical cofactor of PHD [59]. Under these circumstances, the activity of PHD is limited, which indirectly strengthens HIF-1α activation and stabilization, thus triggering overexpression of target genes related to proliferation, cell migration and tumor invasion as clarified above.

### Succinate receptor and its signaling effects

G protein-coupled receptor-91 (GPR91), also termed succinate receptor 1 (SUCNR1), is an orphan molecule that belongs to the G protein-coupled receptor (GPCR) family [60], which was first spotted in a megakaryocytic cell in 1995 and called “P2U2” [61]. The gene encoding GPR91 was later uncovered through an expressed sequence tag data mining strategy in 2001 [62]. GPR91 consists of transmembrane domains connected by three hydrophilic extracellular loops (ECLs) and binding pockets [63]. In recent years, succinate was identified as a specific ligand binds to GPR91 thus triggering downstream physiological and pathophysiological cascades.

GPR91 exhibits a wide distribution and high expression in kidney [64], spleen, liver, small intestine [65], cardiomyocyte [66], retinal ganglion cells [67], white adipose tissue [68], hepatic stellate cells [69] and even on dendritic cells (DCs) [70]. This lays a solid foundation for investigating the role of GPR91 in blood pressure regulation, hematopoiesis [60] and the mechanisms involved in hypertension, heart failure, liver damage, diabetes, and even energy metabolism, angiogenesis and immunomodulation.

Apart from these non-carcinogenic process, succinate also signals as an angiogenesis factor in tumorigenesis. Our recent research revealed that succinate elicits neovascularization by upregulating vascular endothelial growth factor (VEGF) expression in a HIF-independent way, which proves to be through the activation of extracellular regulated kinase (ERK) 1/2 and signal transducer and activator of transcription 3 (STAT3) via the specific succinate receptor GPR91 [22]. An increasing family of evidence suggests that the ERK1/2 signaling pathway is associated with angiogenesis [71], proliferation, differentiation, apoptosis and oncogenesis [65]. In addition, recent findings have indicated that STAT3 is a major oncogenic contributor in diverse cancers, including colon carcinoma [72]. Once stimulated by the accumulation of succinate, the downstream activation will break out immediately, therefore leading to biochemical events and even tumorigenesis.

### TRAP1 expression and SDH inhibition

TRAP1 is an evolutionarily conserved chaperone of the heat shock protein 90 (Hsp90) family, and has been shown to be upregulated in hepatocellular carcinoma [73], gastric cancer [74], colorectal cancer [75], breast cancer [76], thyroid carcinoma [77] and esophageal squamous cell cancer [78]. Recent studies have reported that TRAP1 can decrease SDH enzymatic activity in the respiratory chain, thus resulting in high concentrations of succinate [29, 79, 80]. The accumulated succinate will then contribute to oncogenesis by HIF-1α stabilization, ROS production and GPR91 stimulation, as described above. In addition to the succinate-dependent effect, TRAP1 can also promote cellular migration and invasion through the STAT3/MMP2 pathway and antioxidant defenses [78].

### SDH mutation and NRF2 pathway activation

Nuclear related factor 2 (NRF2) is a transcription factor belonging to the family of nuclear factor erythroid 2-related derived factors (NRFs). NRFs are regulated by Kelch-like ECH-associated protein 1 (KEAP1) and are well-known for their cellular antioxidant defense role in neuron protection [81], liver protection [82] and tumor suppression [83]. However, in recent researches, an evil side of NRF2 gradually surfaced involving in the pathogenesis of various types of tumors [84, 85], such as skin cancer, esophageal cancer, lung cancer [86], and ovarian cancer [87]. Although the evidence of the relationship between SDH mutation and NRF2 activation is insufficient, SDH inhibition is more likely to induce NRF2 production [88], and this may depend on increased ROS production.

### Tumor-associated inflammation

Tumor-promoting inflammation is now an emerging hallmark of cancer, which sounds unanticipated and paradoxical but proved to be virtual in its tumorigenesis journey. Succinate is widely accepted as an inflammatory signal and induces interleukin-1β (IL-1β) through HIF-1α [89] while IL-1β has been demonstrated to be elevated in colorectal cancer [90], oral cancer [91] and colon cancer [92]. Taken together, accumulation of succinate in the tumor microenvironment finally enhances the tumor-associated inflammation. Conversely, inflammation can lead to the release of bioactive molecules, including proangiogenic factors that sustain nutrition supplement, growth factors that support proliferative signaling, and extracellular matrix-modifying enzymes that facilitate angiogenesis, metastasis and invasion [93].

**Figure 2:**
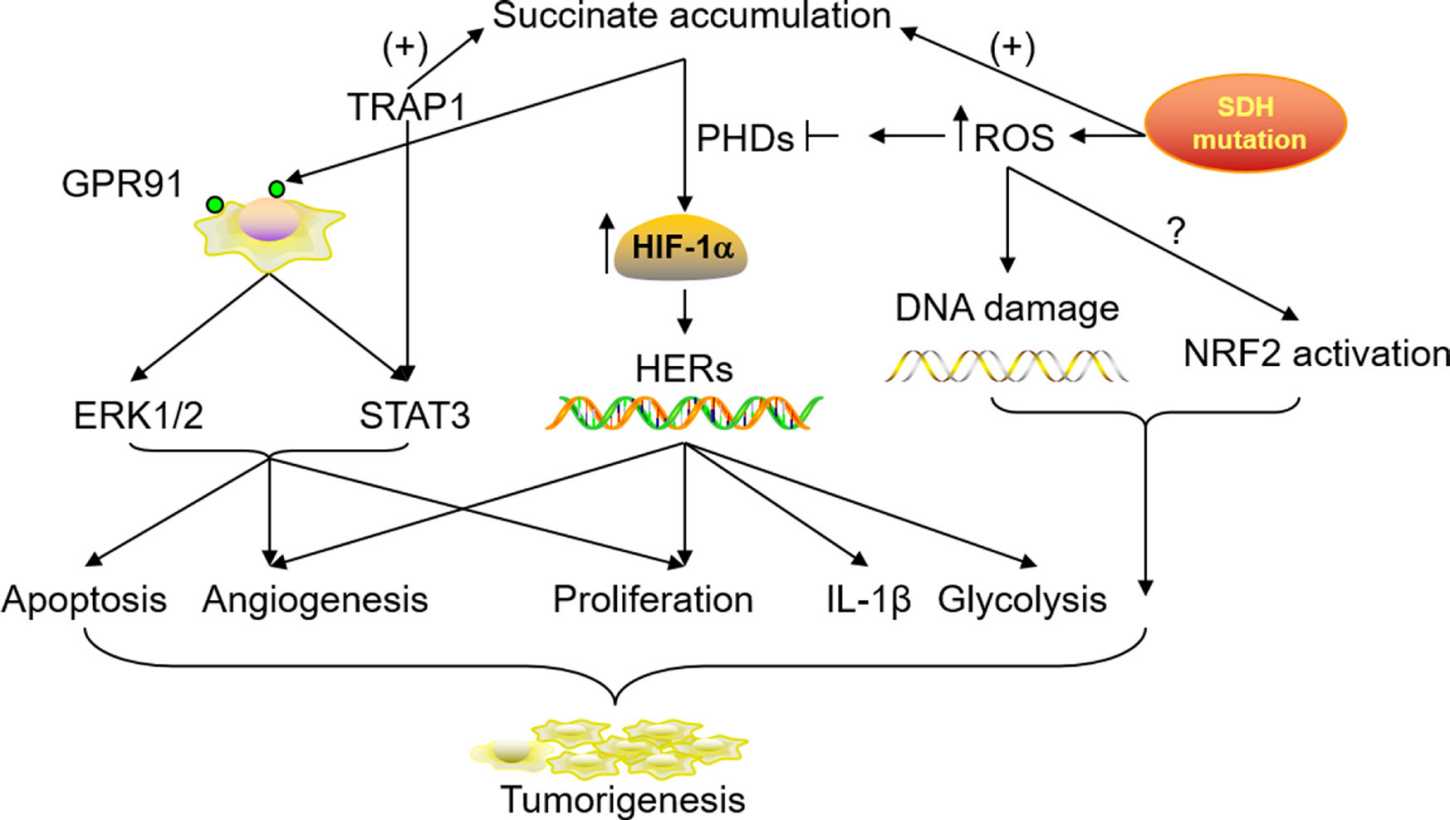
Roles of accumulated succinate in tumorigenesis and progression SDH mutation and TRAP1 up-regulation in the tumor microenvironment can lead to a high concentration of succinate in the cytoplasm, which subsequently results in HIF stabilization, GPR91 activation and downstream signaling cascades. SDH mutation in the mitochondria also contributes to ROS production. This conversely inhibits the activity of PHDs which facilitate HIF catabolism while the accumulated ROS causes DNA damage and NRF2 activation. The involvement and collaborations of all these possible mechanisms promote cell apoptosis, proliferation and migration in tumor tissues.

In summary, all the possible mechanisms responsible for tumorigenesis interact during the multistep of cancer development. The collaborations among mitochondrial function remodeling, HIF stabilization, ROS production, GPR91 activation, TRAP1 expression, NRF2 pathway activation and tumor-associated inflammation via abnormally accumulated succinate can indeed widen the window of a pivotal role for succinate in the acquisition and progression of tumor characteristics (Figure 2).

### Therapeutic targeting

Mechanism-based targeted therapies expand our horizons to treat human tumors. In this study, we cite several therapies that have been applied in the clinic or are currently under development. Angiogenesis plays a key role in the supply of sufficient energy and nutrients for tumors. Antiangiogenic therapies such as VEGF signaling inhibition have been introduced into the ward, but the clinical responses latterly attest to be transitory [94]. TRAP1 is a driver of early stage cancer *in vivo* and the down-regulation of TRAP1 promises to be an “actionable” therapeutic target [31]. ERK1/2 and STAT3 are activated in the signaling pathways and deciphering the mechanism of transcriptional activity may provide a novel therapeutic target. Additionally, we posit that investigational drugs targeting HIF-1α catabolism, ROS inhibition, GPR91 stimulation, PHD activity promotion and tumor-promoting inflammation should be developed to improve clinical treatment.

### Outlook

In this review, we attempt to enumerate the genetic and molecular mechanisms responsible for succinate accumulation and its role as an initiator in neoplasm invasion and metastasis. Looking ahead, we still have more questions than answers. While SDH mutation is generally acknowledged as the culprit for the high level of succinate, the comprehensive mechanisms that contribute to its accumulation remain mysterious. This may suggest a breakthrough point for clinical therapy. Equally, as the specific ligand of GPR91, the precise role of succinate in tumors needs to be explored further, and there may conceal other downstream signaling pathways in addition to STAT3 and ERK1/2 activation. What confuses and attracts us most is the tumor-associated inflammation, whether the tumor-promoting inflammation is different from a common inflammatory process, and the special and specific role of immune cells in tumor-associated inflammation needs further discussion.

## Abbreviations

SDH: succinate dehydrogenase
TCA cycle: tricarboxylic acid cycle
PGL/PCC: paraganglioma/pheochromocytoma
GPR91: G protein-coupled receptor-91
TRAP1: tumor necrosis factor receptor-associated protein 1
FADH2: flavin adenine dinucleotide 2
ICL: isocitrate lyase
HIF-1: Hypoxia inducible factor 1
VDAC/porin: voltage-dependent anion channel
pVHL: von Hippen-Lindau
PHD: prolyl hydroxylases
HREs: HIF response elements
ROS: reactive oxygen species
ECLs: extracellular loops
DCs: dendritic cells
VEGF: vascular endothelial growth factor
ERK1/2: extracellular regulated kinase1/2
STAT3: signal transducer and activator of transcription 3
NRF2: nuclear related factor 2
KEAP1: Kelch-like ECH-associated protein 1
IL-1β: interleukin-1β
Hsp 90: heat shock protein 90.
